# To switch or not? Effects of spokes-character urgency during the social app loading process and app type on user switching intention

**DOI:** 10.3389/fpsyg.2023.1110808

**Published:** 2023-06-07

**Authors:** Ning Zhang, Hsin-Li Hu, Scarlet H. Tso, Chunqun Liu

**Affiliations:** ^1^College of Management, Shenzhen University, Shenzhen, China; ^2^School of Communication, Hang Seng University of Hong Kong, Hong Kong, China; ^3^School of Hotel and Tourism Management, The Chinese University of Hong Kong, Hong Kong, China

**Keywords:** spokes-character urgency, social app orientation, user switching intention, bodily emotional expression, perceived waiting time

## Abstract

Users of mobile phone applications (apps) often have to wait for the pages of apps to load, a process that substantially affects user experience. Based on the Attentional Gate Model and Emotional Contagion Theory, this paper explores the effects of the urgency expressed by a spokes-character’s movement in the loading page of a social app the app type on users’ switching intention through two studies. In Study 1 (*N* = 173), the results demonstrated that for a hedonic-orientated app, a high-urgency (vs. low-urgency) spokes-character resulted in a lower switching intention, whereas the opposite occurred for a utilitarian-orientated app. We adopted a similar methodology in Study 2 (*N* = 182) and the results showed that perceived waiting time mediated the interaction effect demonstrated in Study 1. Specifically, for the hedonic-orientated (vs. utilitarian-orientated) social app, the high-urgency (vs. low-urgency) spokes-character made participants estimate a shorter perceived waiting time, which induces a lower user switching intention. This paper contributes to the literature on emotion, spokes-characters, and human–computer interaction, which extends an enhanced understanding of users’ perception during loading process and informs the design of spokes-characters for the loading pages of apps.

## 1. Introduction

The increasing adoption of smartphones has led to a sharp rise in the number and popularity of mobile phone applications (apps) (Zhao et al., 2016), especially social media apps (Hsiao et al., 2016). Besides, according to the report (eMarketer 2018), users who use social media may increase to 3.29 billion in 2022, occupying 42.3% proportion of the whole world population. Most of the current researches focus on the social app’s hedonic attribution. Hsiao et al. (2016) indicated that the hedonic motivation of the social app is crucial for users to continue use this kind of app. However, more and more social apps also are being used as a way for enterprises to search information, such as Zhihu. Moreover, enterprises use social app to advertise and sell goods. The social app is not only as a hedonic social media now, but also can be considered as a functional product. Akdim et al. (2022) proposed that the social apps both have hedonic and utilitarian attribution. Thus, considering the attribution of social app and giving a appropriate classification is important, which can make social app’s orientation more specific and helpful for their company.

As social media apps are a kind of interactive media, they not only can communicate with others (people, government or enterprises), such as Tik Tok (Hsiao et al., 2016), but also facilitate real-time interaction between the smart phone and the user (Van Noort and Van Reijmersdal, 2019), which has led to these apps becoming closely integrated into users’ daily lives. Consequently, mobile phone users have become sensitive about micro-interactions, which are detailed interactions between the mobile phone and the user (Chen and Li, 2020), especially those that provide visual feedback (Saffer, 2013). A frequently encountered form of micro-interactions is animations that indicate the loading of an app’s page when the network is slow or the loading file is too large, which makes user experience waiting. For instance, we can notice that there is a rotation animation on the APP loading page when using the QQ APP to scan the web page as a result of slow network speed. Users tend to experience negative emotions when they perceive that the loading time of an app exceeds a certain expected threshold (Zhang et al., 2019), which may have detrimental effects on user experience and lead to undesirable consequences such as driving away user traffic, draining users’ mental energy, and reducing companies’ revenue (Kaasinen et al., 2009; Lee et al., 2015; Xie et al., 2021). Therefore, the use of appropriate animations that can minimize users’ perceptions of their waiting time during the loading of pages in apps is critical not only for users, but also for enterprises.

Notably, there are kinds of animations in loading pages, such as spinner animations, progress bar or progress pie animations, figure or cartoon animations, and combination animations (Cao and Hu, 2018; Chen and Li, 2020; Wang et al., 2021). According Chen and Li (2020), using a company’s spokes-character instead of a bar or pie animation on the loading page in an app can reduce users’ perceived waiting time. Previous work examining the use of spokes-characters for this purpose has focused on the effects of either the type of the animations used during the loading process or the actual loading time on users’ satisfaction, emotional experience, perceived waiting time, and threshold for tolerable waiting time (Cao and Hu, 2018; Zhang et al., 2019; Chen and Li, 2020; Wang et al., 2021; Willermark et al., 2021). User interface designers have increasingly used dynamic spokes-characters in loading pages to improve user experience (e.g., in the Huya and Bilibili apps). However, can all dynamic spokes-characters bring the same positive impact?

Research has indicated that individuals’ body movements and postures convey information about their emotions (Wallbott, 1998; Scherer, 2001; Atkinson et al., 2004, 2007; Roether et al., 2009; Dael et al., 2012; Riemer et al., 2017). We identified two kinds emotions state expressed by dynamic spokes-characters that have been used during page loading in apps. The first is the “high-urgency” spokes-character. For instance, in the Huya app, an interactive live broadcasting platform used mainly for the broadcasting of games, the loading page shows an animation in which the app’s spokes-character is flying fast, making it seem as if it is urgent to load during the loading process. The second is the “low-urgency” spokes-character. For instance, in the Bilibili app, a cultural community and video platform with high concentration of young generations in China, the loading page shows two female cartoon characters, girl 22 and girl 33, sitting in a leisurely manner, making them appear relaxed.

This work focused on micro-interactions between users and social apps during the loading process and was grounded on research that has demonstrated that apps are used for a specific purpose, which could be either hedonic or utilitarian in nature. Previous work has roughly categorized brand apps as either informational or entertaining (Bellman et al., 2011; Van Noort and Van Reijmersdal, 2019). In this paper, we further introduced a contextual variable into social app classification, the orientation of a social app, as a potential moderating factor because we posited that users use an app for either chasing recreation (in hedonic-orientated apps) or completing a task (in utilitarian-orientated apps). We conjectured that during the app loading process, the effect of the spokes-character’s urgency may vary depending on the orientation of the app. Our primary research questions were the following: Is there an interaction effect between the spokes-character’s urgency and the social app’s orientation on users’ switching intention, and if so, what are the mechanisms underlying this interaction effect?

To answer these questions, we conducted two laboratory experiments to explore the effects of the spokes-character’s urgency and social app type on the user’s switching intention. Study 1 demonstrated that for a hedonic-orientated social app, a high-urgency (vs. low-urgency) spokes-character was more effective at limiting users’ switching intention, whereas for a utilitarian-orientated social app, a low-urgency (vs. high-urgency) spokes-character was more effective at limiting users’ switching intention. Study 2 empirically probed the mechanisms underlying the interaction effect observed in Study 1 and found that it was mediated by perceived waiting time. That is, for the hedonic-orientated (utilitarian-orientated) app, the high-urgency (vs. low-urgency) spokes-character during the loading progress would make participants perceived a shorter waiting time and generate a lower switching intention.

This paper makes contributions to the literature. First, our work proposes a further categorization of social apps into hedonic-orientated and utilitarian-orientated categories. Previous only a few studies have mainly concentrated on broadly classifying apps, such as into social, game, and productivity apps (Peng et al., 2018). However, with social apps increasingly forming a major proportion of the apps available overall and playing an important role in marketing and product sales (Gulbahar and Yildirim, 2015), a further classification of social app types is necessary, because different kinds of app should have specific designing strategies. Thus, our work may facilitate a deeper understanding of the utility that social apps bring to users. Second, this work extends the literature on emotion by investigating the effect of the spokes-character’s urgency on users’ switching intention for the two types of social apps. The spokes-character’s urgency is expressed through its movements rather than its facial expressions. Most of the literature related to emotion is based on facial expressions, and only a few studies have considered the emotion conveyed by people’s body motion. In the loading pages of social apps, most of the spokes-characters’ emotions are conveyed through body movements because their facial expressions occupy only a small space on the screen. And during this process, the dynamic spokes-characters’ movement capture users’ more attention rather than spokes-characters’ expression. By exploring the effect of the spokes-character’s urgency, a kind of emotion, which is expressed through its movements, this work enriches the literature on emotion in this domain. Third, this research enriches the literature on spokes-characters. Previous studies on spokes-characters have examined the effects of various features of static spokes-characters such as personality (Callcott and Alvey, 1991; Aaker et al., 2004; Garretson and Burton, 2005; Folse et al., 2012, 2013; Phillips et al., 2019), facial completeness and other image design aspects (Zhang et al., 2020), and anthropomorphizing (Zhou et al., 2019; Jeong and Kim, 2021). Moreover, the visual factor (dynamic spokes-characters) dominates users experience during loading process (Wang et al., 2021; Yang et al., 2022). In this loading process, the user submits the request to access the interface firstly, and then the computer response to this demand (loading page with visual feedback, dynamic spokes-character), and simultaneously users see and perceive this feedback. Thus, this work focus the app loading process (computer) and investigate how users (human) perceive and respond to this process, which provides more understanding on human-computer interaction between the devices and users.

In this work, we investigated the effect of the spokes-character’s urgency based on a dynamic spokes-character strategy in the visual design of the loading pages of apps. Our work thus provides a theoretical basis and guidance for the design of app loading pages. In summary, this research contributes both to the literature (on social app categories, bodily expression of emotion, and dynamic spokes-character design) and to practice (in the domain of user interfaces for app loading pages).

## 2. Literature review and hypothesis development

### 2.1. Social media app orientation

Social media refers to a kind of online communication tools, enabling users to share own opinions and exchange information with others on online (Cook and Hopkins, 2008; Kaplan and Haenlein, 2010). And the social media application is the internet-based application (e.g., Facebook app, Tik Tok app), a kind of social media with the development of mobile internet technology, and can present user-generated content (Go and You, 2016). Some researches focus on social media app type. For instance, according to perceptions of the self-presentation and the social presence that the social media app can bring Kaplan and Haenlein (2010), (Go and You, 2016) divide social media apps into six categories (blogs, collaborative projects, social networking sites, content communities, virtual social world, and virtual game worlds). Besides, Some scholars (Kietzmann et al., 2011) believe the social media apps can be classified by their function: identity expression, conversation communication, opinion sharing, presence distance, relationships, reputation, and groups (Go and You, 2016). Most of existing researches on social media app classification based on its social attribution, a few scholars focus on its product attribution. In the context of products, consumers can distinguish between utilitarian and hedonic goods based on whether they have utilitarian or hedonic attributes (Mano and Oliver, 1993; Dhar and Wertenbroch, 2000). Both the hedonic and utilitarian attributes of a product indicate the benefits that it can bring to consumers (Holbrook and Hirschman, 1982; Babin et al., 1994), and a significant amount of research has focused on various product types and their benefits. However, only a few studies have roughly explored the categorization of apps based on the benefits that the brand provide users with (Van Noort and Van Reijmersdal, 2019). Entertainment apps, such as entertainment-orientated video apps, provide users with experiential, hedonic, and entertainment value. Information apps, such as banking apps, meet users’ functional, utilitarian, and informational needs (van Reijmersdal et al., 2010; Van Noort and Van Reijmersdal, 2019). In addition, according to Bellman et al. (2011), brand apps can be categorized as informational/user-centered style (the internal focus) apps and experiential (the external focus) apps and investigate the effect of different brand apps on the interest in the brand and also the brand’s product category. The research (Bellman et al., 2011) focus brands and explore differences of the brand app type on personal connection with the brand, attitude and purchase intention toward the brand, but does not specifically to distinguish these two kinds of apps from the app’s hedonic or utilitarian attribution. Our work would more explicitly divide social media app into two categories from the perspective of the properties of the application, hedonic-orientation or utilitarian-orientation apps. In our study, we posited that social apps too have utilitarian or hedonic characteristics. Hedonic-orientated social apps are designed primarily to bring happiness and pleasant emotional experiences to users and individuals can share or discuss their views with others, whereas utilitarian-orientated social apps are designed primarily to provide practical and functional value and people can find useful information or method to deal with their work or tasks. Furthermore, hedonic-orientated social apps are used for emotional or sensory pleasure and enjoyment, whereas the use of utilitarian social apps is cognitively driven, instrumental, and goal-orientated, as they help users to complete a functional task. Therefore, in this work, we discriminated between hedonic-orientated and utilitarian-orientated social apps to arrive at a nuanced understanding of the effect of the orientation of social apps on users’ experience during the app loading process.

### 2.2. Spokes-character urgency

Since the 1880s, several companies have adopted spokes-characters, such as Michelin’s “Mr. Michelin,” as one of their promotional strategies. A spokes-character is an advertising icon that is used widely for product identification and commercial promotion (Callcott and Alvey, 1991; Garretson and Niedrich, 2004; Zhang et al., 2020) and helps to build a unique brand identity. The use of personable spokes-characters in advertisements or on product packages help to convey a brand’s attributes or brand personality to consumers, thereby fostering favorable impressions and evaluations on consumers (Callcott and Phillips, 1996; Stafford et al., 2002; Garretson and Burton, 2005; Folse et al., 2013; Phillips et al., 2019). In addition, some studies have demonstrated that the personality of a spokes-character can help defend a brand from negative views or mistakes (Aaker et al., 2004; Folse et al., 2013). Some studies on spokes-character strategies have also examined the effect of anthropomorphism. These studies have found that anthropomorphizing money promoted donation intention and the number of donations (Zhou et al., 2019), anthropomorphizing products enhanced purchase intention (Aggarwal and McGill, 2007), and anthropomorphizing brands promoted positive brand attitudes among consumers (Aggarwal and McGill, 2012; Puzakova and Aggarwal, 2018).

Most of these studies have focused on the effects of static spokes-characters’ personality on brand identity, brand equity, brand attitude, and brand defense. However, there is a paucity of studies on dynamic spokes-characters and their emotion in specific situations. A growing body of research has clearly shown that body movement plays a vital role in emotional communication (Dael et al., 2012; De Gelder and Solanas, 2021). Therefore, we posited that the emotion communicated through spokes-characters’ movements in apps is becoming increasingly significant. Our research focused on the effect of spokes-characters’ urgency expressed through their movements on users’ switching intention during the app loading process.

According to *Emotional Contagion Theory*, emotional contagion is a process that an individual’s or a group’s emotion or behavior affects another person’s or group’s feelings or behavioral attitudes whether consciously or unconsciously (Schoenewolf, 1990, p. 50). Thus, individuals have the tendency to automatically emulate and synchronize with the movements, expressions, postures, and sounds of another person that they observe (Hatfield et al., 1993; Riemer et al., 2017). Moreover, recent researches extend *Emotional Contagion Theory* on comparing individuals’ feelings with compatriots, imagining another person’s emotional state, and regarding others’ emotion expression as information (emotional interpretation) (Elfenbein, 2014).

In addition, Hareli and Rafaeli (2008) pointed that, people always have a tendency to recognize and react to the emotions of others. During this process, their personal and affective representations would be activated as then can perceive the states of other individuals (Preston and Hofelich, 2012), extending the range of emotions present (Hareli and Rafaeli, 2008). Thus, when the observer individual and the other person are in the same context, the observer’s emotions may be affected by those of the other person, and when this occurs, the observer is likely to express emotions similar to those of the other person. Especially, Riemer et al. (2017) deem that the bodily expression could be considered as a significant indicator of individual’s emotion. And bodily expression is disparate from facial expression, because the former may be show more complex emotions through kinds of various bodily movement.

Moreover, if the emotion cues provided by the other person’s expressions are consistent with the observer’s emotion, those cues will further induce the observer to have similar experiences or will indirectly motivate the observer to recall similar experiences from the past (Hoffman, 2002). Hence, we posited that a spokes-character that moves quickly with tension during the app loading process, such as by flying or running, may cause a user to recall a related experience, such as running to save time, and therefore lead the user to perceive a sense of urgency of the spokes-character. This perception of urgency may induce the user to think that the spokes-character is in a hurry (high-urgency). In contrast, a spokes-character that appears to be engaged in a leisurely activity, such as playing with a ball, during the app loading process may cause users to think that it is not in a hurry (low-urgency).

In addition, because users typically use hedonic-orientated apps with the expectation of entertainment, they generally do not experience great urgency when the page loads. In such a situation, a high-urgency (vs. low-urgency) spokes-character may reassure a user that the app, visually represented by the spokes-character, is working hard to load the page, and thereby reduce the user’s switching intention. In contrast, because users expect utilitarian-orientated apps to quickly help them achieve their goals, a high-urgency spokes-character may further aggravate a user’s sense of urgency, causing them to experience negative emotions and generating a negative attitude toward the app. Conversely, a low-urgency (vs. high-urgency) spokes-character in utilitarian-orientated apps may alleviate the user’s own sense of urgency, resulting in the positive effect of a reduction in switching intention. Therefore, we hypothesized the following:

*H1a*: In a hedonic-orientated app, a high-urgency (vs. low-urgency) spokes-character results in a lower switching intention among users.

*H1b*: In a utilitarian-orientated app, a low-urgency (vs. high-urgency) spokes-character results in a lower switching intention among users.

### 2.3. Perceived waiting time and attentional gate model

Perceived waiting time refers to the psychological experience of the length of a period of time (Gibbon et al., 1984; Buhusi and Meck, 2005). It is an intuitive feeling and a perceptual ability that people have and use in daily life. Scholars have also referred to the impression of a time interval from tens of milliseconds to tens of seconds as perceived waiting time (Gibbon et al., 1984). In this research, we focused on studying users’ perceived waiting time during the app loading process—that is, users’ perception of the time it took for a page to load. Specifically, the waiting time refers to the duration of loading process and this loading result is not presented. Because we hope to focus on the role of the spokes-character’s urgency during experiencing waiting rather than whether the process success or not.

Perceived waiting time is affected by the extent of a user’s attention to time information. The attentional gate model proposed by Zakay and Block (1996, 1997) can be used to explain people’s perception of time. It consists of three main components: the pacemaker, attentional gate, and working memory (Zakay and Block, 1996, 1997; Branaghan and Sanchez, 2009). The pacemaker generates pulses at a particular rate, and this rate is influenced only by arousal (Zakay and Block, 1997; Branaghan and Sanchez, 2009). The more aroused that the individual is, the quicker the pacemaker pulses. The attentional gate, which represents the individual’s attention, opens wider when the individual is concerned about time, and this concern induces more pulses that are counted and sent to working memory (Zakay and Block, 1997; Buhusi and Meck, 2006). Conversely, the gate closes tighter when the individual pays attention to other objects or ignores temporal stimuli (Zakay and Block, 1997; Branaghan and Sanchez, 2009), resulting in fewer pulses that are counted and sent to working memory. Working memory stores a representation of the elapsed time based on the number of pulses it receives. That is, the more the pulses it receives, the longer it considers the elapsed time to be.

Additionally, Thomas and Weaver (1975) also explains the perception of time. It posits that a human has a limited capacity for attention and that this attention is allocated between two processors in the human information processing system that determine the judgment of a duration: a timer processor (which processes time-related information) and a stimulus processor (which encodes other types of information pertaining to a stimulus). Zakay (1989) suggested that these processors activate together (Thomas and Weaver, 1975). Because of the limited capacity for attention, the perception of the length of a time interval is determined by the ratio of the attention allocated to the two processors. When more resources are devoted to time-related information, the attention allocated to the timer is more than that allocated to the stimulus processor, causing the estimate of a time interval to be relatively long. When fewer resources are devoted to time-related information, the individual’s perception of a time interval is relatively short (Thomas and Weaver, 1975; Zakay, 1989). Hence, users’ attention to time-related information may affect their perceived waiting time during the app loading process.

When waiting for the hedonic-orientation or utilitarian-orientation social app loading, individuals’ motivation, emotion and focal point may be different. Using the hedonic-orientated app, users’ main purpose is to spend their time for pleasure and without time pressure (relaxed emotion). Thus, they would not pay much attention on the waiting time during the app loading process. Accordingly, individuals usually use the utilitarian-orientation app to complete a specific task efficiently in a short time (urgent emotion), hence, they may focus the waiting time when experiencing the app loading. In addition, based on *Emotional Contagion Theory,* the high-urgency or low-urgency spokes-characters expressing by bodily movement on the loading pages can affect users’ emotion. And when waiting for app loading, users own emotion, relaxed or tensive, also should be considered, because individuals can draw attributions and extract specific meanings from spokes-character’s urgency (Hareli and Rafaeli, 2008).

Therefore, we put forward that, when people experience the hedonic-orientation app loading, they pay attention to what presents (the dynamic spokes-character) on the page rather than the waiting time. And in this process, a high-urgency (vs. low-urgency) spokes-character may lead the stimulus processor to become more efficient than the timer processor. This may help the user who use a hedonic app to notice that the high-urgency spokes-character with tensive emotion is making an effort to load the app quickly and thereby reduce switching intention. In contrast, when waiting for the utilitarian-orientation app loading, a high-urgency spokes-character may have a detrimental effect on users perceptions. Because a user with tensive emotion is likely to be eager to obtain information or to reach a specific goal as soon as possible when using a utilitarian-orientated app, the high-urgency spokes-character also with high tension emotion may would induce the timer processor more strongly than the stimulus processor, which leads users focus on waiting time, intensifying the tension of the waiting process. Thus, they may perceive the waiting time is long. Besides, during the utilitarian-orientation app loading, a low-urgency (vs. high-urgency) spokes-character (relaxed emotion) may activate the stimulus processor more strongly than the timer processor, as the low-urgency spokes-character distracts the user’s attention from time and makes users perceive the waiting time is short, which can bring a lower switching intention. Therefore, we hypothesized the following:

*H2a*: In a hedonic-orientated app, a high-urgency (vs. low-urgency) spokes-character reduces the perceived waiting time, which results in a lower switching intention among users.

*H2b*: In a utilitarian-orientated app, a low-urgency (vs. high-urgency) spokes-character reduces the perceived waiting time, which results in a lower switching intention among users.

## 3. Study

### 3.1. Pretest of spokes-character urgency and app orientation

In the studies that we conducted in this work, the spokes-character’s urgency and the app orientation were manipulated using animated images and textual descriptions, respectively. Before conducting the main studies, we performed a pretest with a 2 (spokes-character urgency: high vs. low) by 2 (app orientation: hedonic vs. utilitarian) between-subjects design to ensure that these manipulations were successful.

#### 3.1.1. Stimulus

In this work, we created a video app called Bean Sprouts with a spokes-character called Little Bean Sprouts. The app’s orientation was conveyed to the participants using a research method description from the literature (Crowley et al., 1992). The details of how this was done are provided in Appendix A. To capture the spokes-character’s urgency, we used Photoshop 6.0 and Macromedia Fireworks 8.0 to design app loading GIF images in which the urgency of the spokes-character varied. Specifically, the high-urgency spokes-character was shown flying, conveying a sense of urgency, whereas the low-urgency spokes-character was shown playing with a hula hoop, conveying a relaxed state.

#### 3.1.2. Procedures and measures

One hundred and thirty-four undergraduate students (90 women, *M* age = 20.03 years) from Shenzhen University in China were recruited to participate in the pretest and were randomly assigned to one of four groups based on the 2 × 2 study design. First, we introduced the fictitious Bean Sprouts video app to the participants. The definition of app orientation and a description of the app’s function (see Appendix A) were then presented to the participants, and they were asked to assess the app’s orientation on a 7-point scale (1 = hedonic, 7 = utilitarian). Next, we told participants to imagine: you are using the app and would enter a page that you interest (hedonic-orientation app) or need (utilitarian-orientation app). Due to slow internet, you must wait for the page loading. And next page, you would see the loading process. Then, they were presented with one of two versions of the loading page, with a high-urgency or a low-urgency spokes-character, for 4 s. The participants then evaluated the spokes-character’s urgency on a 7-point scale (1 = low-urgency, 7 = high-urgency). In addition, to control for the effect of the spokes-character’s personality, the participants reported the spokes-character’s likability (Mize and Kinney, 2008; α = 0.90) and expertise (Garretson and Niedrich, 2004; α = 0.89). These personality characteristics were measured based on three items each over a 7-point scale (1 = strongly disagree, 7 = strongly agree). The items are provided in Appendix B. Finally, demographic information was gathered from the participants.

#### 3.1.3. Manipulation check

To check whether our manipulations of the apps’ orientation and the spokes-characters’ urgency were successful, we performed two one-way analyses of variance (ANOVAs) in which the spokes-character urgency and app orientation were the dependent variables, respectively. There was a significant difference between the utilitarian-and hedonic-orientated app groups [*F*(1,133) = 41.62, *p* < 0.001]. The data showed that participants in the utilitarian-orientated app group reported a higher utility score than those in the hedonic-orientated app group (*M*_utilitarian-orientated_ = 4.72, *SD* = 1.48; *M*_hedonic-orientated_ = 2.97, *SD* = 1. 65). The effect of spokes-character urgency was also significant [*F*(1,133) = 40.49, *p* < 0.001]. The participants presented with the high-urgency spokes-character assessed the spokes-character as having a higher urgency (*M*_high-urgency_ = 3.78, *SD* = 1.46) than those presented with the low-urgency spokes-character (*M*_low-urgency_ = 2.36, *SD* = 1.11) during the loading progress. Therefore, our manipulations of the spokes-character’s urgency and the app’s orientation were successful.

#### 3.1.4. Control variables

To make sure the possibility that our manipulations of the spokes-character’s urgency and app orientation do not affect the participants’ assessment of the spokes-character’s personality characteristics, we conducted four additional one-way ANOVAs in which the spokes-character’s likability and expertise were the dependent variable, respectively. The results indicated that our manipulations did not have a significant effect on the participants’ assessment of the spoke-characters’ likability or expertise (*p*s > 0.05).

### 3.2. Study 1

In this study, we sought to verify our hypothesis that in a hedonic-orientated app, the use of a high-urgency (vs. low-urgency) spokes-character during the app’s loading process causes a lower switching intention among users, whereas the opposite holds for utilitarian-orientated apps. We manipulated the app orientation and spokes-character’s urgency to attest the interaction effect of the spokes-character’s urgency during the app loading process and the app orientation on the app switching intention.

#### 3.2.1. Procedures and measures

We adopted a 2 (spokes-character urgency: high vs. low) by 2 (app orientation: hedonic vs. utilitarian) between-subjects design. In total, 173 Chinese (42.8% women; more than 70% participants in the group of 18 and 32 years old) consumers form a Chinese online platform, Wenjuanxing^1^, participated in and they gained ¥3 after completing the study. The procedures used to manipulate the spokes-character’s urgency and the app’s orientation, the questions used to verify whether our manipulations were valid, and the duration for which the loading page lasted were the same as in the pretest. Three items adopted from Davidow (2000) were used to measure the participants’ switching intention based on a 7-point scale (e.g., “I will probably not use this app again”). The items are provided in Appendix B. Finally, we collected the participants’ demographic information and completed the experiment.

#### 3.2.2. Results

##### 3.2.2.1. Validity of manipulations

To check whether our manipulation of the spokes-character’s urgency was successful, we conducted a one-way ANOVA with the participants’ assessment of the spokes-character’s urgency as the dependent variable. There was a significant difference between the high-urgency and low-urgency spokes-character groups [*F*(1,171) = 18.03, *p* < 0.001]. The participants in the high-urgency group reported the spokes-character as having a significantly higher urgency than those in the low-urgency group (*M*_high-urgency_ = 3.58, *SD* = 1.63; *M*_low-urgency_ = 2.54, *SD* = 1.59). Therefore, our manipulation of the spokes-character’s urgency in the app’s loading page was successful.

Similarly, to verify whether our manipulation of the app’s orientation was successful, another one-way ANOVA was conducted with the participants’ perceived app orientation as the dependent variable. There was also a significant difference on APP orientation between the two groups [*F*(1,171) = 27.71, *p* < 0.001]. The participants in the utilitarian-orientated app group reported a significantly higher utility score than those in the hedonic-orientated app group [*M*_utilitarian-orientated_ = 4.00, *SD* = 1.88; *M*_hedonic-orientated_ = 2.66, *SD* = 1.41]. Therefore, our manipulation of the app’s orientation was successful.

##### 3.2.2.2. Users’ switching intention

**Figure 1:**
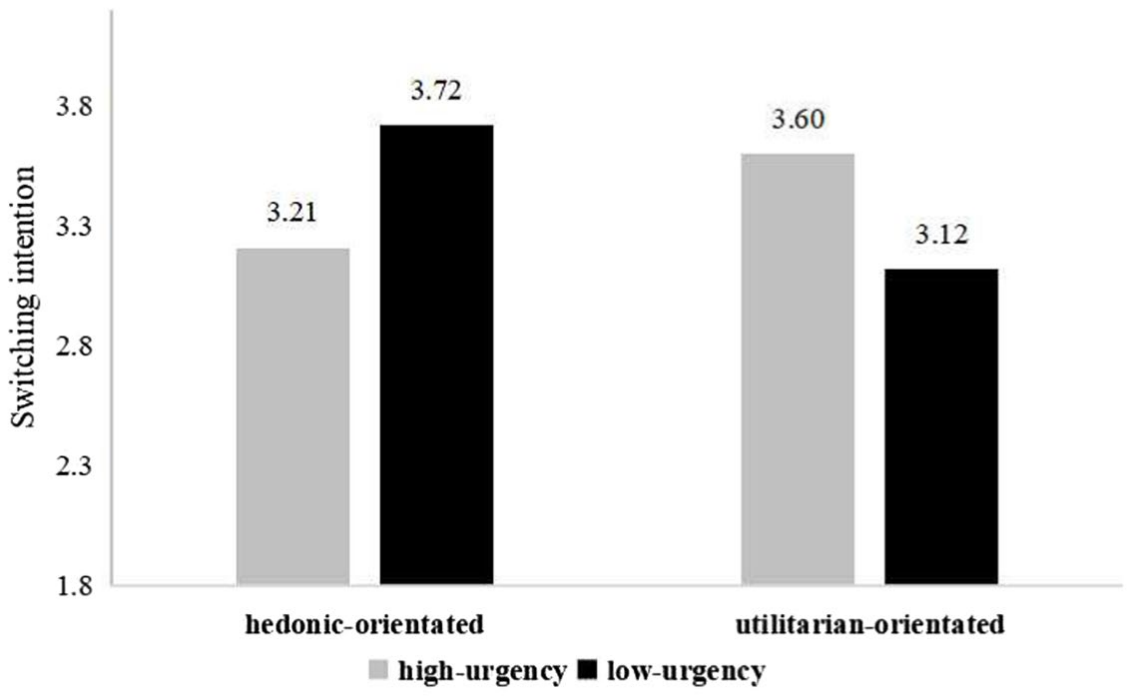
Switching intention (Study 1).

To verify H1a and H1b, a two-way ANOVA was conducted with spokes-character urgency and app orientation as the independent variables and switching intention as the dependent variable. The results revealed that the effect of the interaction between spokes-character urgency and app orientation on switching intention was significant [*F*(1,169) = 9.35, *p* = 0.003]. As shown in the Figure 1, the data analysis indicated that, for the hedonic-orientated app, the high-urgency spokes-character resulted in a lower switching intention than the low-urgency spokes-character [*F*(1,81) = 5.03, *p* = 0. 028; *M*_hedonic-orientated, high-urgency_ = 3.21, *SD* = 0.96; *M*_hedonic-orientated, low-urgency_ = 3.72, *SD* = 1.11]. This result was reversed for the utilitarian-orientated app, as the low-urgency spokes-character resulted in a significantly lower switching intention than the high-urgency one [*F*(1,88) = 4.38, *p* = 0.039; *M*_utilitarian-orientated, low-urgency_ = 3.12, *SD* = 1. 05; *M*_utilitarian-orientated, high-urgency_ = 3.60, *SD* = 1.15].

#### 3.2.3. Discussion

Study 1 confirmed that the effect of the interaction between spokes-character urgency and app orientation on users’ switching intention was significant. The results imply that when an app is used for entertainment, a high-urgency spokes-character in the app’s loading page results in a lower switching intention than a low-urgency spokes-character, whereas when an app is used to perform a specific task, a low-urgency spokes-character leads to a lower switching intention than a high-urgency spokes-character. Therefore, hypotheses H1a and H1b are verified. In Study 2, we further explored the mechanisms underlying these effects by examining the mediating role of perceived waiting time and ruling out other potential alternative pathways.

### 3.3. Study 2

To explore the mechanism underlying the interaction effect between the spokes-character’s urgency and the app’s orientation on users’ switching intention, we measured the perceived waiting time that the participants experienced during the loading of the app’s page. We speculated that the high-urgency (vs. low-urgency) spokes-character would shorten the perceived waiting time in the hedonic-orientated app, which would result in a lower switching intention, and that the opposite would hold for the utilitarian-orientated app.

Moreover, two additional variables, pleasure and arousal, were measured to rule them out as potential alternative mechanisms underlying the effect on switching intention observed in Study 1. Detenber et al. (1998) showed that individuals emotion valence will vary from motion pictures with negative or positive affect. We also posited that the low-urgency (vs. high-urgency) spokes-character causes the participants to experience more delight because its movements reflect the sense that it is leisurely engaged in a joyful game or activity, which may make users feel pleasant and then lead to a lower switching intention Beside, we also posited that the high-urgency (vs. low-urgency) spokes-character creates a stronger visual effect and therefore induces a higher arousal in the participants who use utilitarian-orientation apps, as the rapid movement of the spokes-character appears to be a tensive activity, which may make user feel arousal negative emotion and report a higher switching intention. Thus, we have to rule out the mediation effects of arousal and pleasant in Study 2.

#### 3.3.1. Method

One hundred and ninety participants were recruited from Wenjuanxing, providing a large sample size with qualified participants. Eight of the participants failed to complete the survey, and the final sample therefore comprised completed survey responses from 182 participants (81 women; over 70% between 18 and 32 years old). As in the first study, we adopted a 2 (spokes-character urgency: high vs. low) by 2 (app orientation: hedonic vs. utilitarian) between-subjects design. The participants were randomly assigned to one of the four groups.

##### 3.3.1.1. Procedures and measures

The procedures that were adopted in this study were the same as those in Study 1. The participants first read the definitions of the two types of app orientation. We then introduced a virtual brand, *Panda Reading* (see Appendix C), to the participants and asked them to assess the orientation of the app on a 7-point scale (1 = *hedonic*, 7 = *utilitarian*). Following that, the subjects were told to imagine that they were using the app and were experiencing a loading progress due to a slow network. We presented them with an app loading page with a GIF animation lasting 4 s, immediately after which they were asked to assess the extent of the spokes-character’s urgency, their perceived waiting time (Block, 1990) caused by the loading of the app, and their switching intention. It is possible that the differences in the urgency of the spokes-character between the conditions may have led to differences in the emotional valence (arousal and pleasure) induced in the participants. To test for this potential effect of the spokes-character’s urgency on emotional valence (Angrilli et al., 1997; Balconi and Pozzoli, 2009; Smith et al., 2011), the semantic scales from Kempf (1999) were adopted to measure the degree of pleasure (e.g., unhappy/happy; α = 0.88) and arousal (e.g., sleepy/wake; α = 0.82) experienced by the participants. Finally, the participants’ demographic information, such as age and gender, was gathered.

#### 3.3.2. Results

##### 3.3.2.1. Manipulation checks

To check whether our manipulation of the spokes-character’s urgency was successful, we conducted a one-way ANOVA with the participant’s assessment of the spokes-character’s urgency as the dependent variable. The results showed that the participants in the high-urgency group reported the spokes-character as having a significantly higher urgency than those in the low-urgency group [*F*(1,180) = 29.48, *p* < 0. 001; *M*_high-urgency_ = 3.55, *SD* = 1. 696; *M*_low-urgency_ = 2.30, *SD* = 1.41]. Therefore, our manipulation of the spokes-character’s urgency in the app’s loading page was successful.

To verify whether our manipulation of the app’s orientation was successful, we conducted another one-way ANOVA with the app orientation as the dependent variable. The results indicated that the participants in the utilitarian-orientated app group reported a significantly higher utility score than those in the hedonic-orientated app group [*F*(1,180) = 48.47, *p* < 0. 001; *M*_utilitarian-orientated_ = 4.12, *SD* = 1.92; *M*_hedonic-orientated_ = 2.38, *SD* = 1.35]. Therefore, our manipulation of the app’s orientation was successful.

##### 3.3.2.2. Users’ switching intention

**Figure 2:**
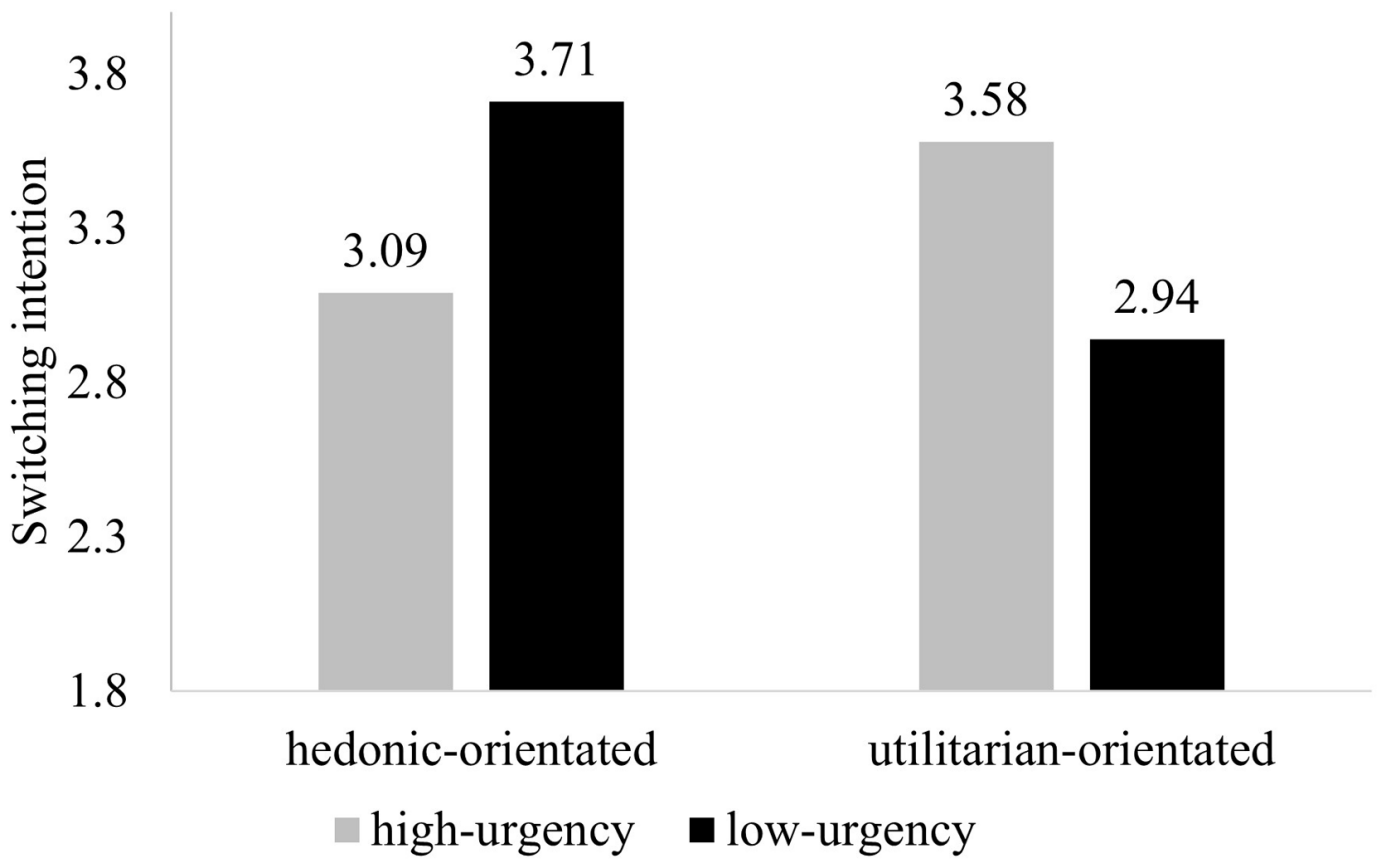
Switching intention (Study 2).

As in Study 1, a two-way ANOVA was performed with spokes-character urgency and app orientation as the independent variables and switching intention as the dependent variable. There was a significant interaction effect (see Figure 2) between spokes-character urgency and app orientation on user switching intention [*F*(1,178) = 18.77, *p* < 0.001]. The results showed that, for the hedonic-orientated app, the high-urgency spokes-character developed a lower switching intention than the low-urgency spokes-character [*F*(1,83) = 7.37, *p* = 0.008; *M*_hedonic-orientated, high-urgency_ = 3.09, *SD* = 1.00; *M*_hedonic-orientated, low-urgency_ = 3.71, *SD* = 1.10]. Conversely, for the utilitarian-orientated app, the low-urgency spokes-character resulted in a significantly lower switching intention than the high-urgency spokes-character [*F*(1,95) = 12.00, *p* = 0.001; *M*_utilitarian-orientated, low-urgency_ = 2.94, *SD* = 0.79; *M*_utilitarian-orientated, high-urgency_ = 3.58, *SD* = 1.2]. Therefore, H1a and H1b were supported again.

##### 3.3.2.3. Perceived waiting time

**Figure 3:**
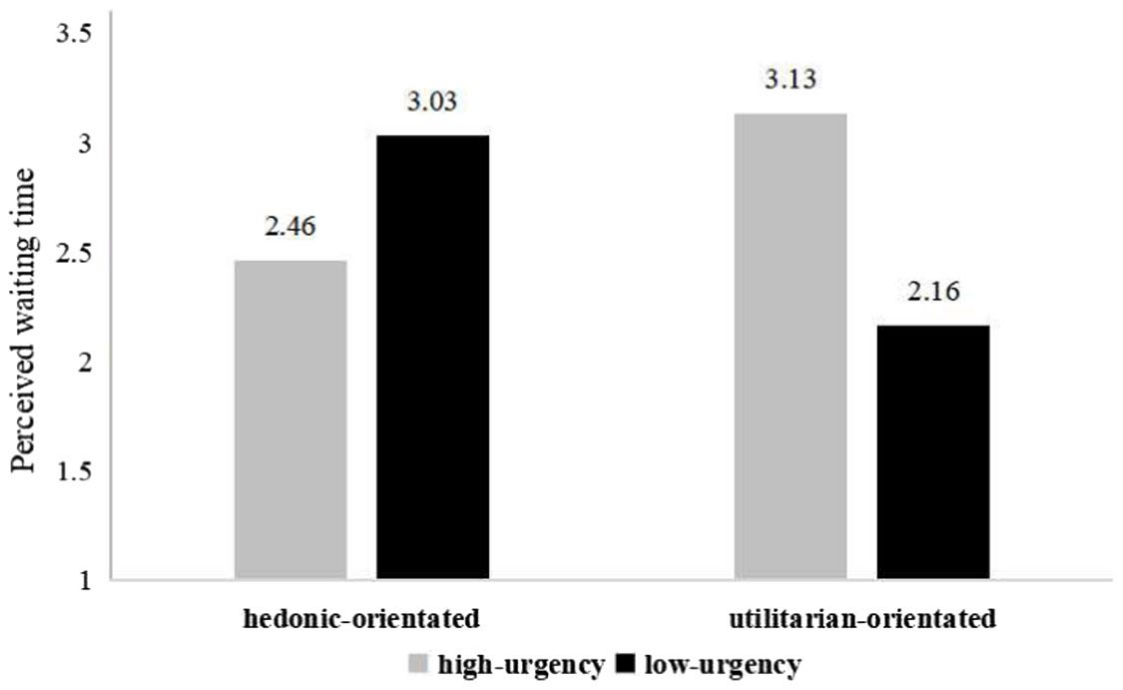
Perceived waiting time (Study 2).

We conducted another two-way ANOVA to test H2a and H2b. This analysis showed that the interaction between spokes-character urgency and app orientation had a significant effect (see Figure 3) on perceived waiting time [*F*(1,178) = 27.14, *p* < 0.001]. The results indicated that, for the hedonic-orientated app, the high-urgency (vs. low-urgency) spokes-character caused participants to report a shorter perceived waiting time than the low-urgency spokes-character [*F*(1,83) = 7.58, *p* = 0. 007; *M*_hedonic-orientated, high-urgency_ = 2.46, *SD* = 0.88; *M*_hedonic-orientated, low-urgency_ = 3.03, *SD* = 1.02]. But for the utilitarian-orientated app, the low-urgency spokes-character induced a shorter perception of the waiting time than the high-urgency spokes-character [*F*(1,95) = 21.57, *p* < 0. 001; *M*_utilitarian-orientated, low-urgency_ = 2.16, *SD* = 0.86; *M*_utilitarian-orientated, high-urgency_ = 3.13, *SD* = 1.19].

##### 3.3.2.4. Alternative mechanisms

A two-way ANOVA was conducted to eliminate the possibility that the observed effects on switching intention were mediated by pleasure or arousal. The independent variables in this test were spokes-character urgency and app orientation, and the dependent variables were arousal and pleasure. The results indicated that there was no main effect of spokes-character urgency or app orientation on either emotion (*p-*values > 0.05) and that the effects of the interaction between spokes-character urgency and app orientation on pleasure and arousal were not significant (*p*s > 0.05).

##### 3.3.2.5. Moderated mediation analysis

To further assess the mediating role of perceived waiting time, we conducted a moderated mediation analysis following the bootstrapping method (with 5,000 iterations) suggested by Hayes (2013). We used the PROCESS macro for SPSS (Hayes, 2013, Model 8) with spokes-character urgency as the independent variable, app orientation as the moderating variable, perceived waiting time as the mediating variable, and switching intention as the dependent variable. The interaction between spokes-character urgency and app orientation predicted perceived waiting time (*B* = −1.54, *SE* = 0.30, 95% CI = [−2.1300, −0.9597]), which significantly predicted switching intention (*B* = −0.17, *SE* = 0.07, 95% CI = [−0.3104, −0.0238]). Moreover, for the hedonic-orientated app, high-urgency spokes-character had a significant indirect effect on switching intention by decreasing perceived waiting time (*B* = −0.16, *SE* = 0.09, 95% CI = [−0.3410, −0.0082]). In contrast, for the utilitarian-orientated app, low-urgency spokes-character had a significant indirect effect on switching intention by reducing perceived waiting time (*B* = 0.10, *SE* = 0.05, 95% CI = [0.0026, 0.2013]). The results confirmed that the mediating effect of perceived waiting time was significant (*B* = 0.26, *SE* = 0.12, 95% CI = [0.0157, 0.5007]). Hence, H2a and H2b were supported.

## 4. General discussion

In this work, we investigated the effect of the interaction between the spokes-character urgency conveyed by an app’s spokes-character and the app’s orientation on users’ switching intention (Study 1), and we revealed the mechanisms underlying that effect (Study 2). Study 1 showed that for the hedonic-orientated app, the high-urgency (vs. low-urgency) spokes-character caused a lower user switching intention, whereas it was the other way around for the utilitarian-orientated app. Study 2 explored the mechanisms underlying this effect and showed that for the hedonic-orientated app, the high-urgency (vs. low-urgency) spokes-character caused a lower switching intention because the high-urgency (vs. low-urgency) spokes-character lead the participants to perceive that their waiting time was shorter. In contrast, for the utilitarian-orientated app, the high-urgency (vs. low-urgency) spokes-character resulted in a lower switching intention because the low-urgency (vs. high-urgency) spokes-character caused the participants to perceive that the waiting time was shorter.

This research contributes both to the literature and to practice. First, in this work, we captured an emotion, sense of urgency, through body movement in the dynamic spokes-character, demonstrating that urgency can be conveyed through vigorous body movements (Keck et al., 2022) such as running, flying and playing. Therefore, our work enriches the literature on emotion, as prior research on the communication and perception of emotion has predominantly focused on facial (Dael et al., 2012; Liu et al., 2014; Aneja et al., 2016; Minaee et al., 2021) and vocal expressions (Clavel et al., 2008; Dael et al., 2012; Han et al., 2014; Minaee et al., 2021).

Second, with the rapid development of mobile media, enterprises have increasingly been adopting spokes-characters as an element in the interface design of app loading pages. However, only a few studies have examined dynamic spokes-characters (Zhang et al., 2021). Prior research has largely focused on the use of static spokes-character images that link the brand with favorable qualities through the personality of the spokes-character (e.g., likability, professionalism, reliability, and nostalgia) to stimulate positive brand evaluation (Callcott and Alvey, 1991; Callcott and Phillips, 1996; Garretson and Burton, 2005; Folse et al., 2012), promote brand equity (Chang, 2014; Folse et al., 2012), or support brand defense (Aaker et al., 2004; Folse et al., 2013). In our work, we focused on dynamic spokes-characters and the expression of emotion through body movement, which was incorporated into the loading page and influenced user switching intention. In addition, we also considered the context of use of the app by introducing specific social app orientation in our studies according to product type, and we demonstrated an interaction effect between the spokes-character’s urgency in the loading page and the orientation of the app. Companies can leverage these findings in the design of their app’s user interface.

Finally, our work focuses on the social app loading process, as the spokes-characters urgency are expressed by their movement and shows on the app page (mobile device), users (human) will be influenced by these spokes-character and generate perceptions about the loading process and the app, thus, this work contributes to the domain of human-computer interaction, which provides insights into understanding deeply how the dynamic spokes-character affect users perceptions during the app loading.

Nevertheless, this research has certain limitations. First, urgency is just one of the attributes of a spokes-character in a loading page. Future research can explore how the urgency emotion can be expressed by bodily movements. And scholars also can examine how other characteristics of spokes-characters affect users’ attitudes and intentions, such as direction of motion (right-orientation or left-orientation) and speed of movement (fast or slow). In addition, in our study, the participants orally reported their perceived waiting time. Future studies can adopt better or additional methods to record perceived waiting time, such as by asking participants to provide an estimate of their waiting time on an analog scale and then allowing them to reproduce their estimated interval by pushing a button to verify its accuracy (Angrilli et al., 1997). Furthermore, this paper does not consider the effect of the facial emotion of the spokes-character, there may be a mixed emotion (facial and bodily expression, negative and positive emotion) effect on the patience of waiting and user switching intention. Lastly, all data in our work are from China, the results of this research may be different in western countries. As Čeněk and Čeněk (2015) proposed, individuals from different cultures have disparate attention, cognition and understanding of images. Some scholars believe that individuals can be more accurate to recognize and perceive the emotion of persons who are from the same cultural group, compared with perceiving out-group emotion expressors (Elfenbein and Ambady, 2002). Our stimulus design is suit for Chinese to recognize and perceive, and the individuals from other culture may perform differently.

## Data availability statement

The original contributions presented in the study are included in the article/Supplementary materials, further inquiries can be directed to the corresponding author.

## Ethics statement

Ethical review and approval was not required for the study on human participants in accordance with the local legislation and institutional requirements. Written informed consent from the participants’ legal guardian/next of kin was not required to participate in this study in accordance with the national legislation and the institutional requirements.

## Author contributions

NZ, CL, H-LH, and ST contributed to the conception and design of the study and wrote sections of the manuscript. CL organized the database. NZ and ST performed the statistical analysis. CL and NZ wrote the first draft of the manuscript. All authors contributed to manuscript revision, read and approved the submitted version.

## Funding

The authors are grateful for the financial support from the National Natural Science Foundation of China (Grant Numbers: 71402100, 71772127, and 71832015).

## Conflict of interest

The authors declare that the research was conducted in the absence of any commercial or financial relationships that could be construed as a potential conflict of interest.

## Publisher’s note

All claims expressed in this article are solely those of the authors and do not necessarily represent those of their affiliated organizations, or those of the publisher, the editors and the reviewers. Any product that may be evaluated in this article, or claim that may be made by its manufacturer, is not guaranteed or endorsed by the publisher.

## Footnotes

^1^ https://www.sojump.com/
