# Supplementary material for: To switch or not? Effects of spokes-character urgency during the social app loading process and app type on user switching intention
Source: Front Psychol. 2023 Jun 7;14:1110808. doi: 10.3389/fpsyg.2023.1110808 (PMC10299737; doi:10.3389/fpsyg.2023.1110808)
Supplement: Supplementary file 1 [file Data_Sheet_1.doc]

**Appendix A - The description of APP orientation (Pretest and Study 1)**

*Manipulation of APP orientation*

**Step 1: Giving the definition of the two APP orientation to participants.**

*In daily life, mobile social apps can be divided into hedonistic-oriented apps and utilitarian-oriented apps. The hedonistic-oriented APP can make user feel emotional and sensory pleasant (Tik Tok). And utilitarian-oriented apps can be used to achieve a goal or complete a practical task (Zhihu).*

**Step 2: The introduction of the APP**

***The hedonistic-oriented APP is described as:***

*“Bean Sprouts” is a hedonistic-oriented movie recommendation and sharing APP, which provides love movies, comedy movies, action movies, science fiction movies etc, to provide users with audio-visual enjoyment, as well as emotional and sensory dual pleasant. And this APP also shows reviews of every movie and users can talk about movies in this APP.*

***The utilitarian-oriented APP is described as:***

*“Bean Sprouts” is an utilitarian-oriented movie recommendation sharing APP which provides a large number of documentary films and public information or data for users to understand the evolution of humans and the environment in the historical process. And this APP also shows opinions of every movie and users can talk about movies in this APP.*

**Step 3: Showing the loading process to participants (see Appendix C)**

**Step 4: Measuring variables, asking manipulation questions and collecting demographic information**

| **Variables** | **Measures** | **Reference** |
| --- | --- | --- |
| likability | I think the spokes-character is   1. Cute 2. Favorable 3. Pleasant | Callcott & Alvey, (1991) |
| expertise | 1. I thing the spokes-character likes an expert. 2. I think the spokes-character is experienced. 3. I think the spokes-character is knowledgeable. | Garretson & Niedrich, (2004) |
| switching intention | 1. I will use this APP much less in the future. 2. I will probably switch to another APP in the future. 3. I will probably not use this APP again. | Davidow, (2000) |
| arousal | Please rate your emotion   1. sleepy/wake 2. relaxed/stressed 3. calm/excited | Kempf, (1999) |
| pleasant | Please rate your emotion   1. unhappy/happy 2. angry/pleasant 3. unsatisfied/satisfactory | Kempf, (1999) |

**Appendix B- Items**

**Appendix C - The description of APP orientation (Study 2)**

*Manipulation of APP orientation (Same to Study 1)*

**Step 1: Giving the definition of the two social APP orientation to participants.**

*In daily life, mobile social apps can be divided into hedonistic-oriented apps and utilitarian-oriented apps. The hedonistic-oriented APP can make user feel emotional and sensory pleasant (Tik Tok). And utilitarian-oriented apps can be used to achieve a goal or complete a practical task (Zhihu).*

**Step 2: The introduction of the APP**

***The hedonistic-oriented APP is described as:***

*"Panda Reading" is a hedonic-oriented social APP that provides users with a large number of reading resources for various types of novels, including ancient romance novels, love stories, modern fictions, fantasy novels and so on. Users can talk about their feeling about the novel by using this APP.*

***The utilitarian-oriented APP is described as:***

*“Panda Reading” is a utilitarian-oriented social APP that provides users with a large number of literary novels at home and abroad, such as A Dream of Red Mansions, Border Town, War and Peace, and A Year in Provence. Through the APP, users can read literary works and learn about the local customs of different regions from other users.*

**Step 3: Showing the loading process to participants (see Appendix F)**

**Step 4: Measuring variables, asking manipulation questions and collecting demographic information**
